# Crystal structure of ethyl 4-(2-meth­oxy­phen­yl)-6-methyl-2-sulfanyl­idene-1,2,3,4-tetra­hydro­pyrimidine-5-carboxyl­ate

**DOI:** 10.1107/S2056989015010026

**Published:** 2015-06-03

**Authors:** Shaaban K. Mohamed, Joel T. Mague, Mehmet Akkurt, Ahmed Khodairy, Eman A. Ahmed

**Affiliations:** a Chemistry and Environmental Division, Manchester Metropolitan University, Manchester M1 5GD, England; b Chemistry Department, Faculty of Science, Minia University, 61519 El-Minia, Egypt; c Department of Chemistry, Tulane University, New Orleans, LA 70118, USA; d Department of Physics, Faculty of Sciences, Erciyes University, 38039 Kayseri, Turkey; e Chemistry Department, Faculty of Science, Sohag University, 82524 Sohag, Egypt

**Keywords:** crystal structure, Biginelli reactions, dihyro­pyrimidino­nes, three-component reactions, N—H⋯S inter­actions

## Abstract

In the title compound, C_15_H_18_N_2_O_3_S, the hydro­pyrimidine ring adopts a sofa conformation with the methine C atom as the flap. The benzene ring is almost perpendicular to the mean plane of the hydro­pyrimidine ring, making a dihedral angle of 85.51 (8)°, and the meth­oxy O atom lies over the centre of the pyrimidine ring. In the crystal, weak N—H⋯S inter­actions form a zigzag chain running along the *b-*axis direction.

## Related literature   

For syntheses of di­hydro­pyrimidino­nes and their analogous, see: Biginelli (1893 ▸); Varala *et al.* (2003 ▸); Gohain *et al.* (2004 ▸); Ahmed *et al.* (2009 ▸). For biological activities of hydro­pyrimidino­nes, see: Salehi *et al.* (2006 ▸); Singh *et al.* (2010 ▸); Hed *et al.* (2009 ▸); Russowsky *et al.* (2007 ▸); Shah *et al.* (2009 ▸). For the synthesis of the title compound, see: Ahmed *et al.* (2012 ▸).
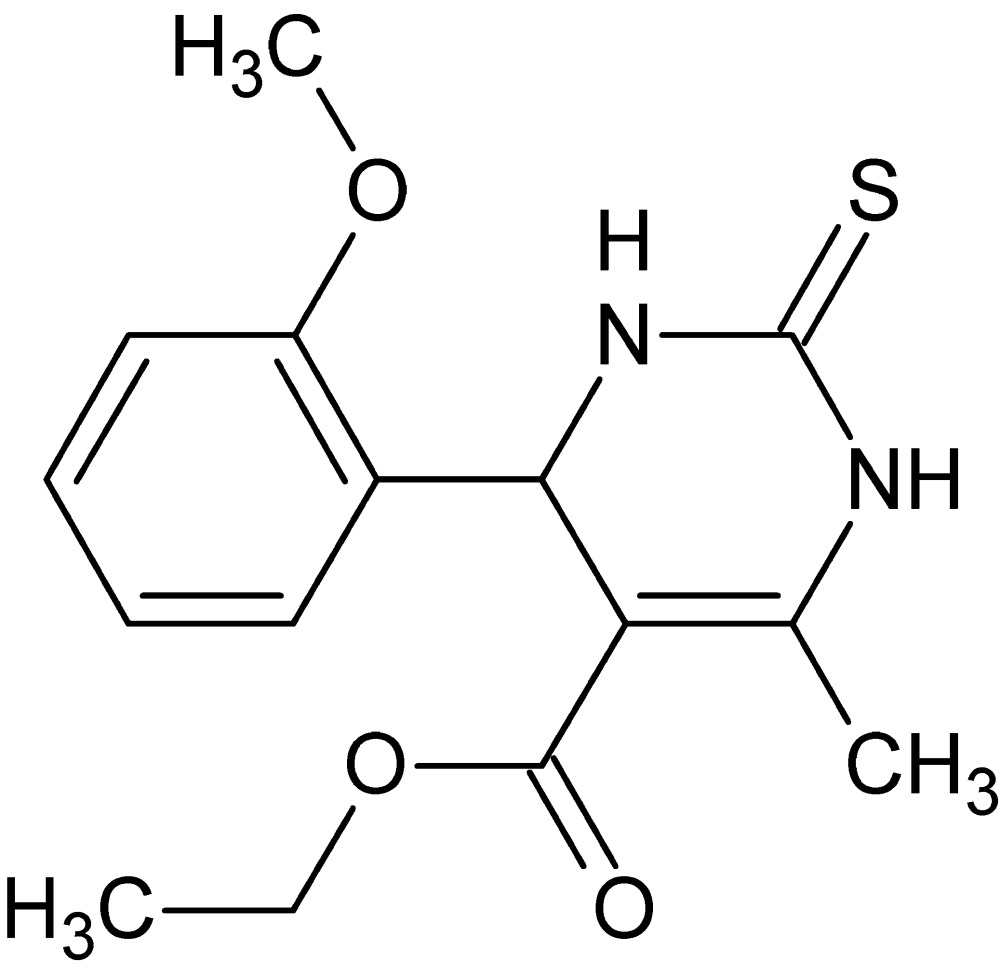


## Experimental   

### Crystal data  

C_15_H_18_N_2_O_3_S
*M* *_r_* = 306.37
Triclinic, 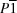

*a* = 7.9791 (2) Å
*b* = 8.2031 (2) Å
*c* = 11.8405 (3) Å
α = 81.987 (1)°
β = 87.975 (1)°
γ = 80.850 (1)°
*V* = 757.60 (3) Å^3^
*Z* = 2
Cu *K*α radiation
μ = 2.00 mm^−1^
*T* = 150 K
0.25 × 0.21 × 0.12 mm

### Data collection  

Bruker D8 VENTURE PHOTON 100 CMOS diffractometer
Absorption correction: multi-scan (*SADABS*; Bruker, 2014 ▸) *T*_min_ = 0.73, *T*_max_ = 0.79
9145 measured reflections
2929 independent reflections
2773 reflections with *I* > 2σ(*I*)
*R*_int_ = 0.021

### Refinement  

*R*[*F* ^2^ > 2σ(*F* ^2^)] = 0.039
*wR*(*F* ^2^) = 0.101
*S* = 1.08
2929 reflections
193 parameters
H-atom parameters constrained
Δρ_max_ = 0.43 e Å^−3^
Δρ_min_ = −0.26 e Å^−3^

### 

Data collection: *APEX2* (Bruker, 2014 ▸); cell refinement: *SAINT* (Bruker, 2014 ▸); data reduction: *SAINT*; program(s) used to solve structure: *SHELXT* (Sheldrick, 2015*a* ▸); program(s) used to refine structure: *SHELXL2014* (Sheldrick, 2015*b* ▸); molecular graphics: *DIAMOND* (Brandenburg & Putz, 2012 ▸); software used to prepare material for publication: *SHELXL2014*.

## Supplementary Material

Crystal structure: contains datablock(s) global, I. DOI: 10.1107/S2056989015010026/is5401sup1.cif

Structure factors: contains datablock(s) I. DOI: 10.1107/S2056989015010026/is5401Isup2.hkl

Supporting information file. DOI: 10.1107/S2056989015010026/is5401Isup3.cml

**Fig. 1.**
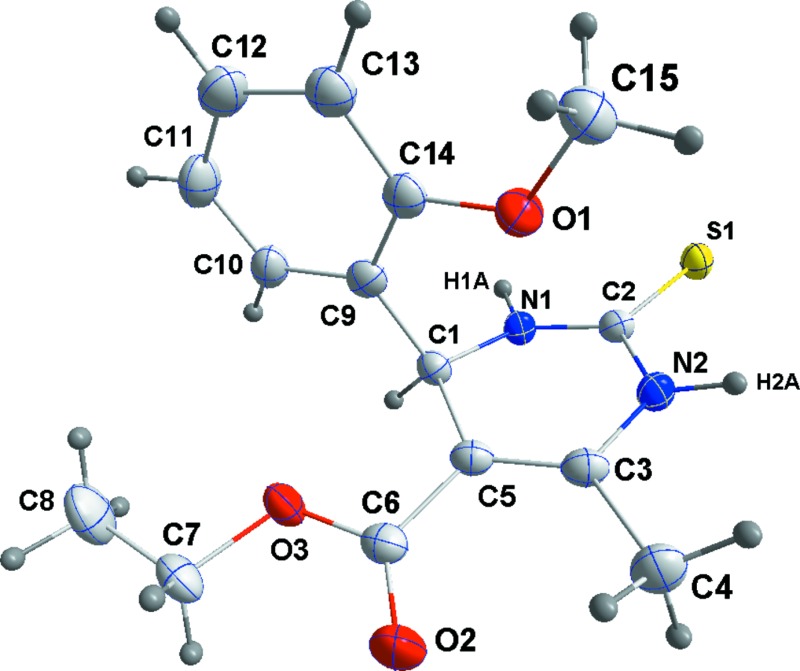
The molecular structure of the title compound showing labeling scheme and 50% probability ellipsoids.

. DOI: 10.1107/S2056989015010026/is5401fig1.tif
The mol­ecular structure of the title compound showing labeling scheme and 50% probability ellipsoids.

**Fig. 2.**
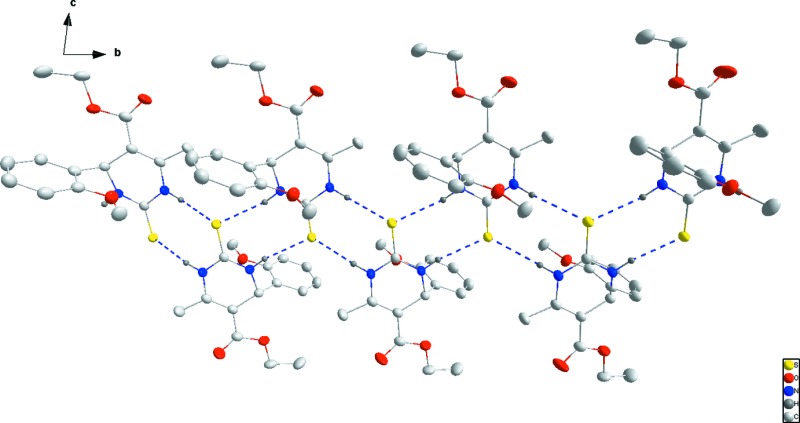
A section of the chain formed by N—H···S hydrogen bonds (dashed lines).

**Fig. 3.**
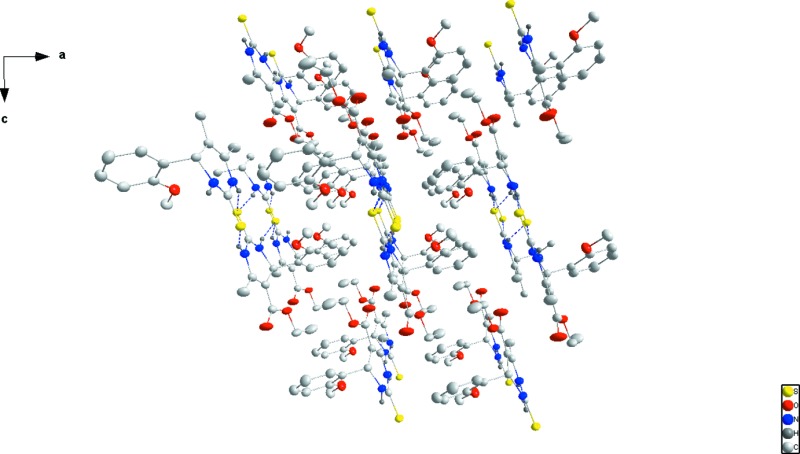
A packing diagram viewed along the b axis. N—H···S interactions are shown as dotted lines.

. DOI: 10.1107/S2056989015010026/is5401fig2.tif
A section of the chain formed by N—H⋯S hydrogen bonds (dashed lines).

b . DOI: 10.1107/S2056989015010026/is5401fig3.tif
A packing diagram viewed along the *b* axis. N—H⋯S inter­actions are shown as dotted lines.

CCDC reference: 1402530

Additional supporting information:  crystallographic information; 3D view; checkCIF report

## Figures and Tables

**Table 1:** Hydrogen-bond geometry (, )

*D*H*A*	*D*H	H*A*	*D* *A*	*D*H*A*
N1H1*A*S1^i^	0.91	2.46	3.3539(13)	167
N2H2*A*S1^ii^	0.91	2.58	3.4327(14)	157

Symmetry codes: (i) 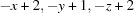; (ii) 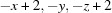.

## supplementary crystallographic information

### S1. Comment

Dihydropyrimidin-2(1*H*)-one scaffold compounds are an important class of substances in organic and medicinal chemistry. Aryl-substituted 3, 4-dihydropyrimidin-2(1*H*)-ones and their sulfur analogue have been reported to possess diverse range of pharmacological activity (Salehi *et al.*, 2006) such as anticancer, anti HIV, antibacterial, antimalarial, antihypertensive, sedative, hypnotics, anticonvulsant, antithyroid,antihistaminic agents and antibiotics (Singh *et al.*, 2010; Hed *et al.*, 2009; Russowsky *et al.*, 2007; Shah *et al.*, 2009). This stimulated the invention of a wide range of synthetic methods for their preparation and chemical transformations. In recent years, several modified procedures have been reported to improve the efficiency of the Biginelli dihydropyrimidine synthesis (Biginelli, 1893) by using different catalysts *e.g.* Lewis acids (Varala *et al.*, 2003; Gohain *et al.*, 2004) or by using basic condition *via* phase transfer catalysis (Ahmed *et al.*, 2009). In this context, we report in this study the crystal structure of the title compound.

In the title compound (Fig. 1), the plane of the benzene ring is almost parallel to the C1···N2 vector with the methoxy oxygen atom (O1) lying over the centre of the pyrimidine ring. The pyrimidine ring has Cremer-Pople puckering parameters Q = 0.201 (2) Å, θ = 62.2 (5)° and φ = 42.9 (2)°. In the crystal, weak N—H···S interactions (Table 1) form a chain running parallel to the *b* axis (Figs. 2 & 3).

### S2. Experimental

The title compound was prepared according to our reported method (Ahmed *et al*., 2012). Colourless crystals suitable for X-ray analysis were grown from ethanol (*m.p.* 473–475 K, yield 98%).

### S3. Refinement

H-atoms attached to C were placed in calculated positions (C—H = 0.95–1.00 Å), while those attached to N were placed in a difference map and their parameters adjusted to give N—H = 0.91 Å. All were included as riding contributions with isotropic displacement parameters 1.2 or 1.5 times those of the attached atoms.

### Crystal data

**Table:**

C_15_H_18_N_2_O_3_S	*Z* = 2
*M**_r_* = 306.37	*F*(000) = 324
Triclinic, *P*1	*D*_x_ = 1.343 Mg m^−^^3^
*a* = 7.9791 (2) Å	Cu *K*α radiation, λ = 1.54178 Å
*b* = 8.2031 (2) Å	Cell parameters from 7936 reflections
*c* = 11.8405 (3) Å	θ = 3.8–72.2°
α = 81.987 (1)°	µ = 2.00 mm^−^^1^
β = 87.975 (1)°	*T* = 150 K
γ = 80.850 (1)°	Thick plate, colourless
*V* = 757.60 (3) Å^3^	0.25 × 0.21 × 0.12 mm

### Data collection

**Table:**

Bruker D8 VENTURE PHOTON 100 CMOS diffractometer	2929 independent reflections
Radiation source: INCOATEC IµS micro–focus source	2773 reflections with *I* > 2σ(*I*)
Mirror monochromator	*R*_int_ = 0.021
Detector resolution: 10.4167 pixels mm^-1^	θ_max_ = 72.2°, θ_min_ = 3.8°
ω scans	*h* = −9→9
Absorption correction: multi-scan (*SADABS*; Bruker, 2014)	*k* = −10→10
*T*_min_ = 0.73, *T*_max_ = 0.79	*l* = −14→14
9145 measured reflections	

### Refinement

**Table:**

Refinement on *F*^2^	Primary atom site location: structure-invariant direct methods
Least-squares matrix: full	Secondary atom site location: difference Fourier map
*R*[*F*^2^ > 2σ(*F*^2^)] = 0.039	Hydrogen site location: mixed
*wR*(*F*^2^) = 0.101	H-atom parameters constrained
*S* = 1.08	*w* = 1/[σ^2^(*F*_o_^2^) + (0.0487*P*)^2^ + 0.4254*P*] where *P* = (*F*_o_^2^ + 2*F*_c_^2^)/3
2929 reflections	(Δ/σ)_max_ = 0.001
193 parameters	Δρ_max_ = 0.43 e Å^−^^3^
0 restraints	Δρ_min_ = −0.26 e Å^−^^3^

### Special details

**Table:**

Geometry. All e.s.d.'s (except the e.s.d. in the dihedral angle between two l.s. planes) are estimated using the full covariance matrix. The cell e.s.d.'s are taken into account individually in the estimation of e.s.d.'s in distances, angles and torsion angles; correlations between e.s.d.'s in cell parameters are only used when they are defined by crystal symmetry. An approximate (isotropic) treatment of cell e.s.d.'s is used for estimating e.s.d.'s involving l.s. planes.
Refinement. Refinement of *F*^2^ against ALL reflections. The weighted *R*-factor *wR* and goodness of fit *S* are based on *F*^2^, conventional *R*-factors *R* are based on *F*, with *F* set to zero for negative *F*^2^. The threshold expression of *F*^2^ > σ(*F*^2^) is used only for calculating *R*-factors(gt) *etc*. and is not relevant to the choice of reflections for refinement. *R*-factors based on *F*^2^ are statistically about twice as large as those based on *F*, and *R*- factors based on ALL data will be even larger. H-atoms attached to carbon were placed in calculated positions (C—H = 0.95 - 0.98 Å) while those attached to nitrogen were placed in locations derived from a difference map and their parameters adjusted to give N—H = 0.91 Å. All were included as riding contributions with isotropic displacement parameters 1.2 - 1.5 times those of the attached atoms.

### Fractional atomic coordinates and isotropic or equivalent isotropic displacement parameters (Å^2^)

**Table:**

	*x*	*y*	*z*	*U*_iso_*/*U*_eq_	
S1	1.08339 (5)	0.22865 (4)	1.02799 (3)	0.02400 (13)	
O1	0.64475 (15)	0.28798 (15)	0.86937 (10)	0.0312 (3)	
O2	0.8568 (2)	0.26940 (18)	0.47910 (11)	0.0494 (4)	
O3	0.75790 (16)	0.52294 (15)	0.52331 (9)	0.0304 (3)	
N1	0.96037 (16)	0.42742 (15)	0.84338 (11)	0.0213 (3)	
H1A	0.9617	0.5109	0.8864	0.026*	
N2	0.99441 (17)	0.14788 (16)	0.83198 (11)	0.0240 (3)	
H2A	1.0080	0.0431	0.8710	0.029*	
C1	0.85934 (19)	0.47576 (19)	0.73835 (13)	0.0216 (3)	
H1	0.9084	0.5685	0.6916	0.026*	
C2	1.00503 (19)	0.27318 (19)	0.89394 (13)	0.0209 (3)	
C3	0.9414 (2)	0.1739 (2)	0.71936 (13)	0.0247 (3)	
C4	0.9585 (3)	0.0158 (2)	0.66684 (15)	0.0351 (4)	
H4A	0.8722	0.0271	0.6082	0.053*	
H4B	0.9429	−0.0770	0.7259	0.053*	
H4C	1.0717	−0.0061	0.6320	0.053*	
C5	0.88282 (19)	0.3296 (2)	0.66990 (13)	0.0230 (3)	
C6	0.8339 (2)	0.3648 (2)	0.54883 (14)	0.0277 (3)	
C7	0.6888 (2)	0.5701 (2)	0.40933 (14)	0.0331 (4)	
H7A	0.6051	0.4980	0.3959	0.040*	
H7B	0.7806	0.5588	0.3512	0.040*	
C8	0.6050 (3)	0.7480 (3)	0.40272 (18)	0.0486 (5)	
H8A	0.5172	0.7580	0.4625	0.073*	
H8B	0.5529	0.7841	0.3278	0.073*	
H8C	0.6900	0.8183	0.4136	0.073*	
C9	0.6761 (2)	0.5437 (2)	0.76670 (13)	0.0255 (3)	
C10	0.6100 (2)	0.7098 (2)	0.72635 (15)	0.0304 (4)	
H10	0.6797	0.7785	0.6817	0.036*	
C11	0.4425 (3)	0.7744 (2)	0.75156 (17)	0.0382 (4)	
H11	0.3972	0.8861	0.7228	0.046*	
C12	0.3434 (2)	0.6753 (2)	0.81827 (16)	0.0370 (4)	
H12	0.2298	0.7205	0.8357	0.044*	
C13	0.4043 (2)	0.5117 (2)	0.86057 (15)	0.0320 (4)	
H13	0.3342	0.4452	0.9068	0.038*	
C14	0.5720 (2)	0.4459 (2)	0.83384 (13)	0.0269 (3)	
C15	0.5435 (2)	0.1762 (2)	0.93170 (16)	0.0361 (4)	
H15A	0.4988	0.2205	1.0013	0.054*	
H15B	0.6133	0.0671	0.9521	0.054*	
H15C	0.4489	0.1642	0.8845	0.054*	

### Atomic displacement parameters (Å^2^)

**Table:**

	*U*^11^	*U*^22^	*U*^33^	*U*^12^	*U*^13^	*U*^23^
S1	0.0304 (2)	0.0195 (2)	0.0222 (2)	−0.00308 (15)	−0.00427 (14)	−0.00340 (14)
O1	0.0296 (6)	0.0277 (6)	0.0355 (6)	−0.0073 (5)	0.0044 (5)	0.0011 (5)
O2	0.0744 (10)	0.0443 (8)	0.0274 (7)	0.0095 (7)	−0.0101 (6)	−0.0165 (6)
O3	0.0391 (7)	0.0314 (6)	0.0209 (6)	−0.0055 (5)	−0.0058 (5)	−0.0026 (5)
N1	0.0255 (6)	0.0180 (6)	0.0217 (6)	−0.0048 (5)	−0.0032 (5)	−0.0046 (5)
N2	0.0309 (7)	0.0181 (6)	0.0238 (6)	−0.0034 (5)	−0.0014 (5)	−0.0056 (5)
C1	0.0237 (7)	0.0218 (7)	0.0202 (7)	−0.0052 (6)	−0.0020 (6)	−0.0033 (6)
C2	0.0202 (7)	0.0206 (7)	0.0227 (7)	−0.0045 (6)	0.0013 (6)	−0.0044 (6)
C3	0.0261 (8)	0.0261 (8)	0.0241 (8)	−0.0069 (6)	0.0019 (6)	−0.0084 (6)
C4	0.0497 (11)	0.0273 (9)	0.0309 (9)	−0.0059 (8)	−0.0026 (8)	−0.0121 (7)
C5	0.0235 (7)	0.0256 (8)	0.0217 (7)	−0.0063 (6)	0.0014 (6)	−0.0067 (6)
C6	0.0278 (8)	0.0332 (9)	0.0233 (8)	−0.0059 (7)	0.0010 (6)	−0.0066 (6)
C7	0.0370 (9)	0.0430 (10)	0.0199 (8)	−0.0104 (8)	−0.0046 (7)	0.0000 (7)
C8	0.0683 (14)	0.0410 (11)	0.0346 (10)	−0.0088 (10)	−0.0160 (10)	0.0062 (8)
C9	0.0268 (8)	0.0292 (8)	0.0222 (7)	−0.0048 (6)	−0.0031 (6)	−0.0081 (6)
C10	0.0335 (9)	0.0276 (8)	0.0289 (8)	0.0007 (7)	−0.0053 (7)	−0.0055 (7)
C11	0.0398 (10)	0.0284 (9)	0.0436 (10)	0.0035 (8)	−0.0066 (8)	−0.0041 (8)
C12	0.0352 (9)	0.0377 (10)	0.0367 (9)	0.0019 (8)	0.0000 (7)	−0.0094 (8)
C13	0.0320 (9)	0.0368 (9)	0.0278 (8)	−0.0069 (7)	−0.0017 (7)	−0.0049 (7)
C14	0.0294 (8)	0.0286 (8)	0.0233 (8)	−0.0044 (7)	−0.0036 (6)	−0.0049 (6)
C15	0.0366 (10)	0.0323 (9)	0.0386 (10)	−0.0106 (8)	0.0047 (8)	0.0033 (8)

### Geometric parameters (Å, º)

**Table:**

S1—C2	1.6969 (15)	C5—C6	1.476 (2)
O1—C14	1.349 (2)	C7—C8	1.498 (3)
O1—C15	1.430 (2)	C7—H7A	0.9900
O2—C6	1.205 (2)	C7—H7B	0.9900
O3—C6	1.340 (2)	C8—H8A	0.9800
O3—C7	1.4541 (19)	C8—H8B	0.9800
N1—C2	1.322 (2)	C8—H8C	0.9800
N1—C1	1.4783 (18)	C9—C14	1.397 (2)
N1—H1A	0.9098	C9—C10	1.403 (2)
N2—C2	1.3580 (19)	C10—C11	1.395 (3)
N2—C3	1.391 (2)	C10—H10	0.9500
N2—H2A	0.9098	C11—C12	1.376 (3)
C1—C5	1.522 (2)	C11—H11	0.9500
C1—C9	1.523 (2)	C12—C13	1.382 (3)
C1—H1	1.0000	C12—H12	0.9500
C3—C5	1.347 (2)	C13—C14	1.403 (2)
C3—C4	1.501 (2)	C13—H13	0.9500
C4—H4A	0.9800	C15—H15A	0.9800
C4—H4B	0.9800	C15—H15B	0.9800
C4—H4C	0.9800	C15—H15C	0.9800
			
C14—O1—C15	118.77 (14)	C8—C7—H7A	110.4
C6—O3—C7	116.45 (13)	O3—C7—H7B	110.4
C2—N1—C1	125.30 (12)	C8—C7—H7B	110.4
C2—N1—H1A	117.1	H7A—C7—H7B	108.6
C1—N1—H1A	114.7	C7—C8—H8A	109.5
C2—N2—C3	123.59 (14)	C7—C8—H8B	109.5
C2—N2—H2A	116.2	H8A—C8—H8B	109.5
C3—N2—H2A	119.5	C7—C8—H8C	109.5
N1—C1—C5	108.79 (12)	H8A—C8—H8C	109.5
N1—C1—C9	110.68 (12)	H8B—C8—H8C	109.5
C5—C1—C9	115.38 (12)	C14—C9—C10	118.77 (16)
N1—C1—H1	107.2	C14—C9—C1	121.76 (15)
C5—C1—H1	107.2	C10—C9—C1	119.46 (15)
C9—C1—H1	107.2	C11—C10—C9	120.32 (17)
N1—C2—N2	117.28 (14)	C11—C10—H10	119.8
N1—C2—S1	122.70 (11)	C9—C10—H10	119.8
N2—C2—S1	119.94 (12)	C12—C11—C10	119.55 (17)
C5—C3—N2	119.62 (14)	C12—C11—H11	120.2
C5—C3—C4	127.50 (15)	C10—C11—H11	120.2
N2—C3—C4	112.89 (14)	C11—C12—C13	121.84 (17)
C3—C4—H4A	109.5	C11—C12—H12	119.1
C3—C4—H4B	109.5	C13—C12—H12	119.1
H4A—C4—H4B	109.5	C12—C13—C14	118.62 (17)
C3—C4—H4C	109.5	C12—C13—H13	120.7
H4A—C4—H4C	109.5	C14—C13—H13	120.7
H4B—C4—H4C	109.5	O1—C14—C9	115.19 (15)
C3—C5—C6	121.63 (14)	O1—C14—C13	123.92 (15)
C3—C5—C1	120.93 (14)	C9—C14—C13	120.89 (16)
C6—C5—C1	117.42 (14)	O1—C15—H15A	109.5
O2—C6—O3	122.34 (16)	O1—C15—H15B	109.5
O2—C6—C5	126.96 (16)	H15A—C15—H15B	109.5
O3—C6—C5	110.70 (13)	O1—C15—H15C	109.5
O3—C7—C8	106.67 (14)	H15A—C15—H15C	109.5
O3—C7—H7A	110.4	H15B—C15—H15C	109.5
			
C2—N1—C1—C5	−25.4 (2)	C3—C5—C6—O3	171.37 (14)
C2—N1—C1—C9	102.32 (16)	C1—C5—C6—O3	−6.8 (2)
C1—N1—C2—N2	16.9 (2)	C6—O3—C7—C8	177.69 (16)
C1—N1—C2—S1	−166.47 (11)	N1—C1—C9—C14	−61.89 (18)
C3—N2—C2—N1	0.7 (2)	C5—C1—C9—C14	62.18 (19)
C3—N2—C2—S1	−176.02 (12)	N1—C1—C9—C10	116.92 (15)
C2—N2—C3—C5	−6.0 (2)	C5—C1—C9—C10	−119.01 (16)
C2—N2—C3—C4	173.96 (15)	C14—C9—C10—C11	−1.1 (2)
N2—C3—C5—C6	176.60 (14)	C1—C9—C10—C11	−179.90 (15)
C4—C3—C5—C6	−3.3 (3)	C9—C10—C11—C12	1.3 (3)
N2—C3—C5—C1	−5.3 (2)	C10—C11—C12—C13	−0.7 (3)
C4—C3—C5—C1	174.72 (16)	C11—C12—C13—C14	−0.3 (3)
N1—C1—C5—C3	18.7 (2)	C15—O1—C14—C9	−175.65 (14)
C9—C1—C5—C3	−106.35 (17)	C15—O1—C14—C13	4.3 (2)
N1—C1—C5—C6	−163.15 (13)	C10—C9—C14—O1	−179.93 (14)
C9—C1—C5—C6	71.79 (18)	C1—C9—C14—O1	−1.1 (2)
C7—O3—C6—O2	5.8 (2)	C10—C9—C14—C13	0.1 (2)
C7—O3—C6—C5	−174.41 (13)	C1—C9—C14—C13	178.93 (14)
C3—C5—C6—O2	−8.9 (3)	C12—C13—C14—O1	−179.41 (16)
C1—C5—C6—O2	172.98 (18)	C12—C13—C14—C9	0.5 (2)

### Hydrogen-bond geometry (Å, º)

**Table:**

*D*—H···*A*	*D*—H	H···*A*	*D*···*A*	*D*—H···*A*
N1—H1*A*···S1^i^	0.91	2.46	3.3539 (13)	167
N2—H2*A*···S1^ii^	0.91	2.58	3.4327 (14)	157

Symmetry codes: (i) −*x*+2, −*y*+1, −*z*+2; (ii) −*x*+2, −*y*, −*z*+2.
