# Supplementary figures and images for: Crystal structure of ethyl 4-(2-meth­oxy­phen­yl)-6-methyl-2-sulfanyl­idene-1,2,3,4-tetra­hydro­pyrimidine-5-carboxyl­ate
Source: Acta Crystallogr E Crystallogr Commun. 2015 Jun 3;71(Pt 7):o444–5. doi: 10.1107/S2056989015010026 (PMC4518927; doi:10.1107/S2056989015010026)

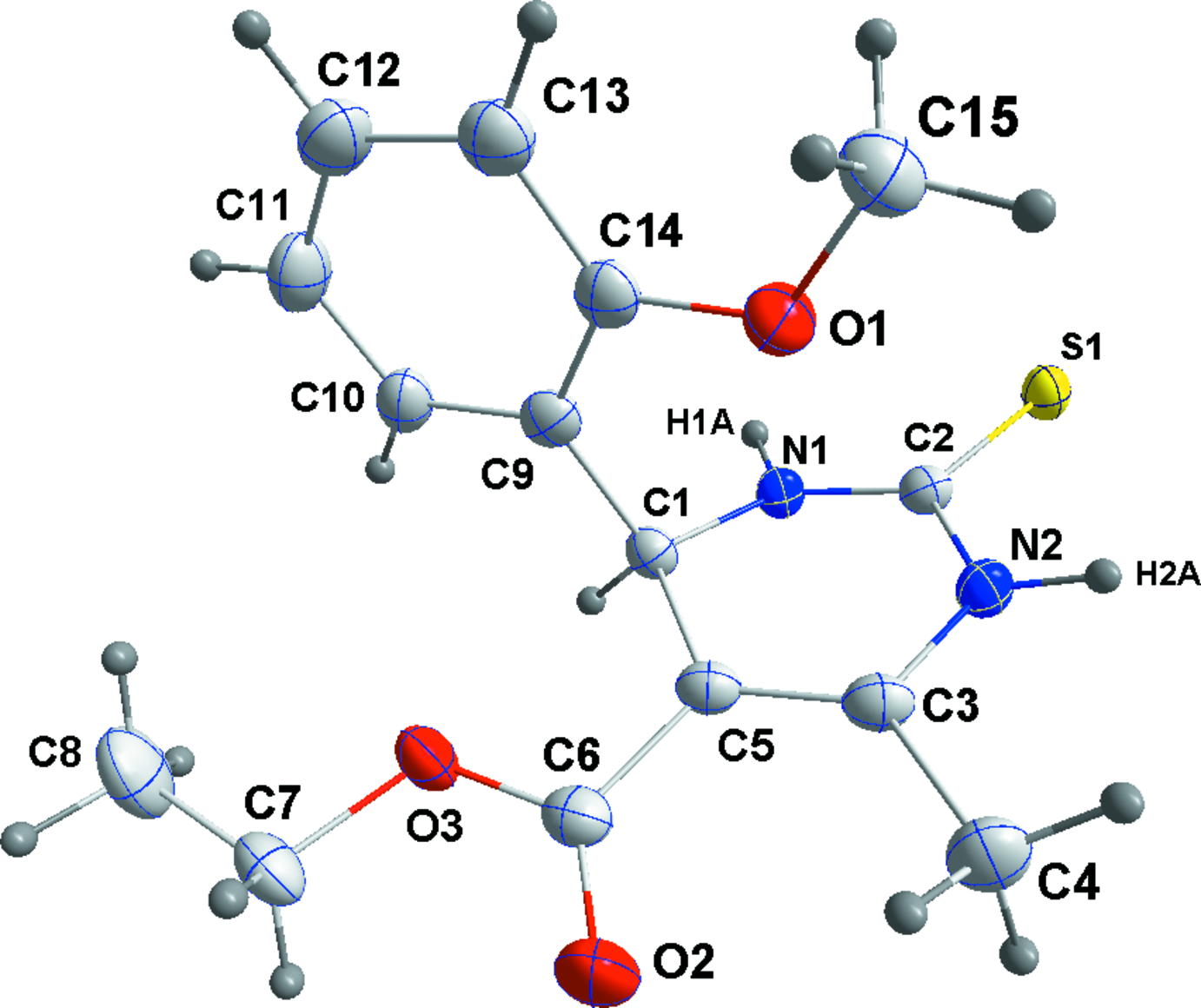

Supplement: Supplementary file 4 [file e-71-0o444-fig1.tif]

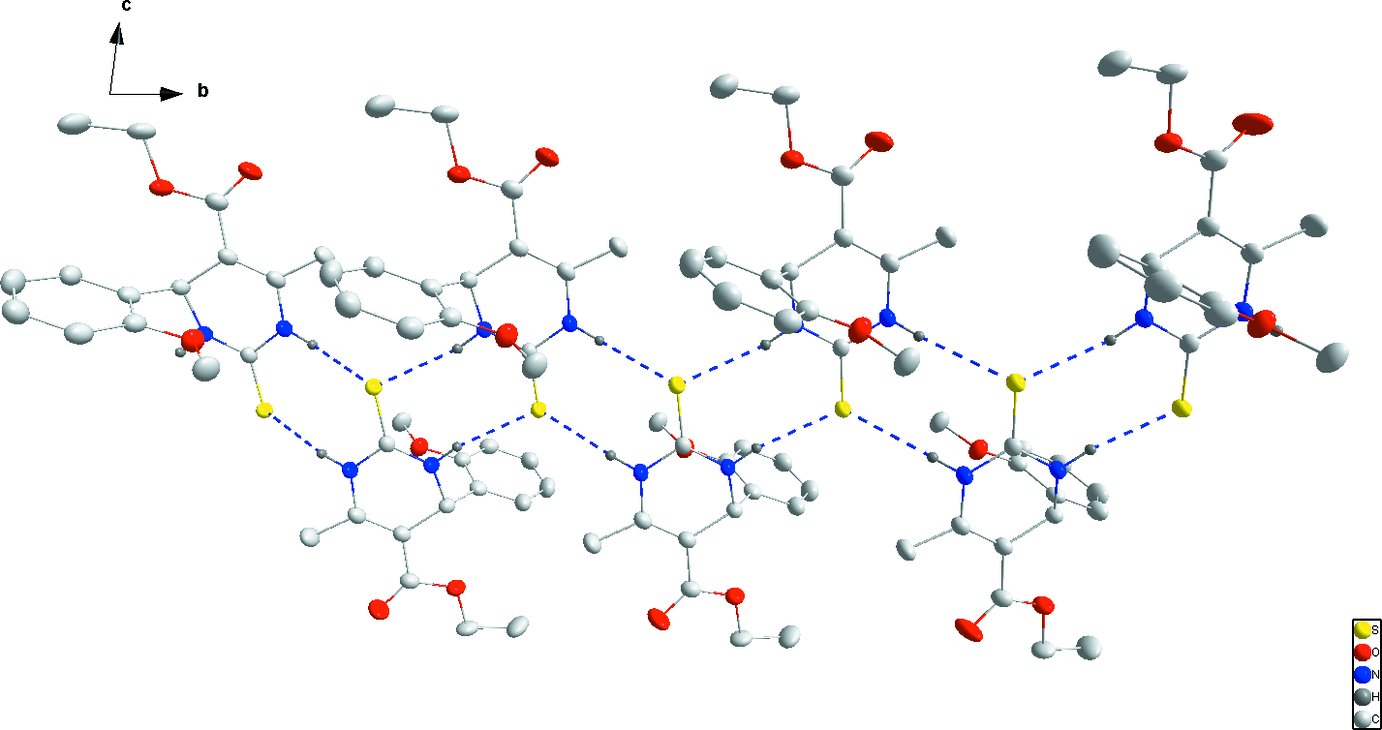

Supplement: Supplementary file 5 [file e-71-0o444-fig2.tif]

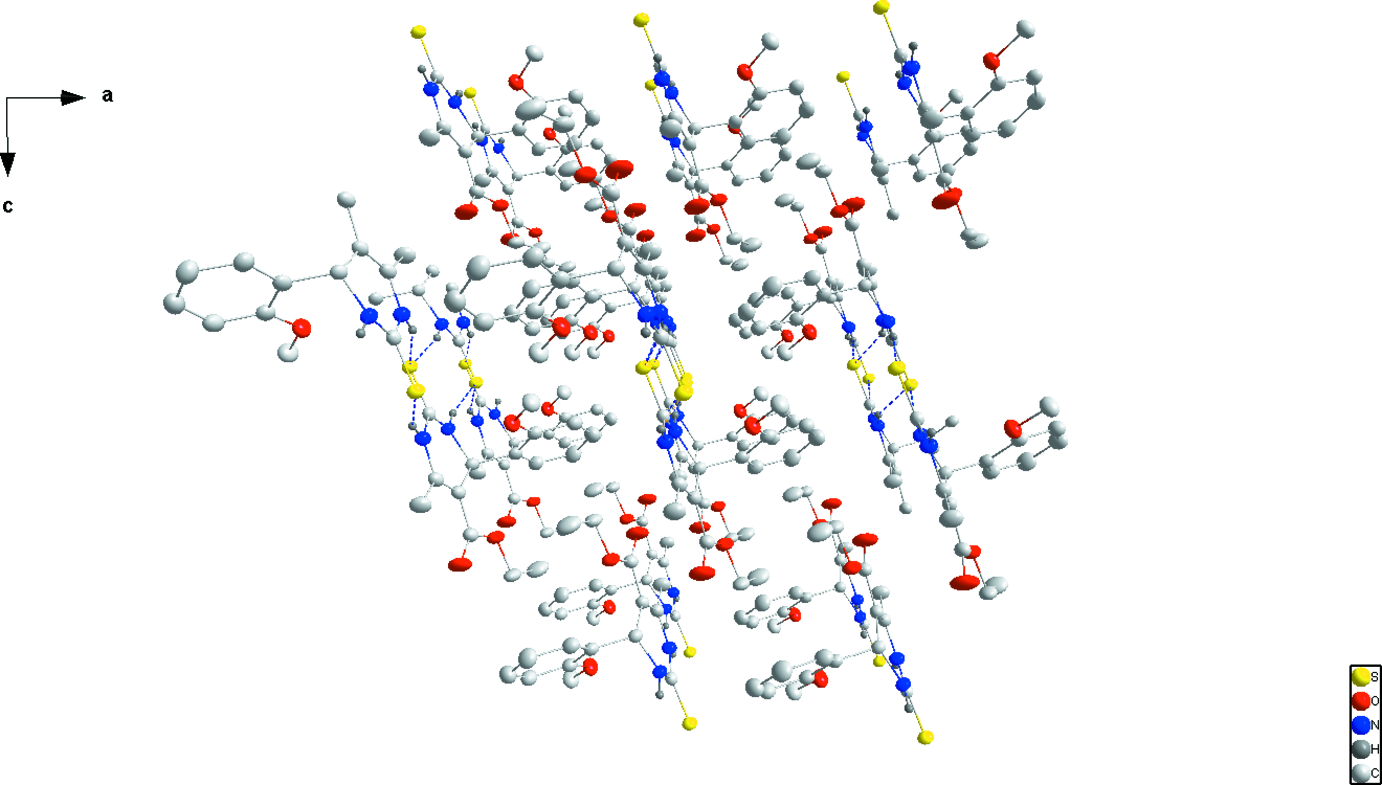

Supplement: Supplementary file 6 [file e-71-0o444-fig3.tif]
